# Healthcare Resource Use Associated With Tumor-Induced Osteomalacia: A Literature Review

**DOI:** 10.1210/clinem/dgae431

**Published:** 2024-06-24

**Authors:** Suzanne M Jan de Beur, Kathryn M Dahir, Erik A Imel, María Belén Zanchetta, Angela Williams, Zhiyi Li, Neil Webb, Victoria Crowe, Ben Johnson, Thomas O Carpenter

**Affiliations:** Division of Endocrinology and Metabolism, Department of Medicine, University of Virginia, Charlottesville, VA 22903, USA; Division of Endocrinology and Metabolism, Department of Medicine, Vanderbilt University Medical Center, Nashville, TN 37232, USA; Departments of Medicine and Pediatrics (Endocrinology), Indiana University School of Medicine, Indianapolis, IN 46202-5111, USA; Instituto de Investigaciones Metabólicas, Universidad del Salvador, Buenos Aires, ZC 1012, Argentina; Department of Health Economics and Outcomes Research, Kyowa Kirin International, Marlow, Buckinghamshire, SL7 1HZ, UK; Department of Health Economics and Outcomes Research, Kyowa Kirin North America, Bedminster, NJ 07921, USA; Systematic Review Department, Source Health Economics, Oxford, OX2 7BY, UK; Systematic Review Department, Source Health Economics, Oxford, OX2 7BY, UK; Department of Health Economics and Outcomes Research, Kyowa Kirin International, Marlow, Buckinghamshire, SL7 1HZ, UK; Departments of Pediatrics (Endocrinology) and Orthopaedics and Rehabilitation, Yale School of Medicine, New Haven, CT 06520-8064, USA

**Keywords:** tumor-induced osteomalacia, healthcare resource, diagnosis, resection, clinical burden

## Abstract

**Context:**

Tumor-induced osteomalacia (TIO) is an ultra-rare, paraneoplastic syndrome caused by tumors that secrete fibroblast growth factor 23 (FGF23). Initial signs and musculoskeletal symptoms can be nonspecific and unrecognized, leading to long delays in diagnosis and treatment, and resulting in severe and progressive disability in patients with TIO.

**Objective:**

This review aimed to identify published evidence on healthcare resource use in TIO to better understand the burden of the disease.

**Evidence acquisition:**

A targeted literature review was conducted to identify publications reporting on disease characteristics and healthcare resource use associated with TIO.

**Evidence synthesis:**

In total, 414 publications were included in the review, of which 376 were case reports. From the case reports, data on 621 patients were extracted. These patients had a mean (SD) age of 46.3 (15.8) years; 57.6% were male. Mean time from first symptoms to diagnosis of TIO was 4.6 (4.7) years and, in cases where imaging tests were reported, patients underwent a mean of 4.1 (2.7) procedures. Tumor resection was attempted in 81.0% of patients and successful in 67.0%. Fracture was reported in 49.3% of patients. Results from association analyses demonstrated that longer time to diagnosis was associated with poorer tumor resection outcomes and a higher probability of tumor recurrence. Unfavorable tumor resection outcomes were associated with greater use of pharmacologic treatment and a greater likelihood of orthopedic surgery.

**Conclusion:**

TIO is associated with a substantial healthcare resource burden. Improvements in the diagnostic process could lead to better management of TIO, thereby benefiting patients and reducing that burden.

Tumor-induced osteomalacia (TIO) is an ultra-rare, paraneoplastic syndrome caused by tumors, often of mesenchymal origin, that secrete fibroblast growth factor 23 (FGF23) (1), a hormone regulating phosphate handling and vitamin D metabolism (2). The excess levels of FGF23 in TIO lead to impaired renal phosphate reabsorption, reduced active vitamin D synthesis, and chronic hypophosphatemia (3). Patients with TIO present with musculoskeletal symptoms that may include bone fractures, musculoskeletal pain, fatigue, and severe myopathy (4, 5). However, initial signs and symptoms can be nonspecific and therefore TIO may remain insidious, leading to long delays in diagnosis (4, 5). These delays are further compounded by the fact that serum phosphorus levels are not routinely measured in clinical practice, and therefore, hypophosphatemia itself is frequently unrecognized (5). Due to its rarity, data on the prevalence and incidence of TIO are scarce, although an epidemiologic study in Denmark reported an incidence rate of 0.13 per 100 000 persons per year (6); a more recent German study infers an incidence rate of 0.094 cases per 100 000 persons per year and a prevalence of 0.187 cases per 100 000 persons (7).

For treatment, complete surgical resection of the causative tumor, when possible, is curative in most cases. However, in 35% to 40% of patients presenting with features consistent with TIO, the tumor cannot be localized (8, 9). Additionally, incomplete resection of the tumor often results in symptomatic persistence and/or recurrence (3). In cases when the tumor cannot be localized or completely resected, treatment with oral phosphate and active vitamin D is recommended (10, 11). However, this treatment can be associated with complications, such as secondary/tertiary hyperparathyroidism, frequent gastrointestinal (GI) distress, hypercalcemia, hypercalciuria, nephrocalcinosis, kidney stones, and renal dysfunction (3, 10). Additionally, treatment with phosphate supplementation can be burdensome, due to its unpleasant taste and the need for frequent administration, often 4 to 6 times a day (10, 12). Furthermore, treatment costs for oral supplementation such as calcitriol are not often covered by insurance (13). Recently, burosumab, a fully human monoclonal antibody which binds to and inhibits the activity of FGF23, has been approved in several countries for the treatment of TIO in cases where the tumor cannot be localized or curatively resected (14-17).

TIO imposes a significant burden on patients and healthcare resources due to the severe and progressive disability experienced by untreated patients (4, 18). Symptoms such as fatigue and musculoskeletal pain severely impact patient health-related quality of life, similar to those symptoms experienced by patients with advanced cancer (4). Additionally, prolonged diagnostic delays due to nonspecific symptomatology and rarity of the condition are typical and contribute further to the high clinical burden of disease associated with TIO (4, 18). With few data available on the resource burden associated with the disease, a targeted literature review was conducted to identify published evidence reporting on the healthcare resource use in TIO, in order to better understand the extent of that burden.

## Methods

A targeted literature review was conducted in electronic databases to identify publications reporting on disease characteristics and healthcare resource use (including investigations, treatments, and clinical events) associated with TIO.

### Eligibility Criteria

The population, intervention, comparator(s), outcomes, and study design (PICOS) elements for the targeted literature review are presented in Table 1.

**Table 1.** Eligibility criteria (PICOS)

Characteristics	Inclusion criteria	Exclusion criteria
Population	People with a diagnosis of TIO	Non-oncogenic associated osteomalacia
Intervention /comparators	No restriction	—
Outcomes	Including but not limited to: Time from onset of symptoms to diagnosis Frequency of tumor investigations (imaging [eg, MRI, CT, radiography, ultrasound, PET scan, octreotide scan], biopsies) Routine management and diagnostic procedures (eg, laboratory investigations [including urine and blood] and healthcare consultations including specialists for misdiagnosed conditions) Attempted surgical resection of tumor % in which resection is successful % in which tumor recurs Pharmacologic treatment (eg, oral phosphate, active vitamin D, cinacalcet, burosumab) Duration of treatment Proportion of patients receiving treatment History of fractures/number and mean number of fractures/pseudo fractures, types of fractures and pseudo fractures Resource use associated with ongoing disease and symptom management: Orthopedic procedures Management of morbidities related to TIO (eg, management of pain, fatigue, physical therapies, and muscle weakness, mental health service use) Management of morbidities typically associated with oral phosphate and active vitamin D treatment (eg, nephrocalcinosis, hyperparathyroidism, parathyroidectomy, gastrointestinal symptoms) Use of mobility aids Costs Productivity loss Absenteeism and presenteeism related to work/school/caregivers Disability and dropping out of the work force	No relevant outcomes reported
Study design	Any studies reporting original cost and/or resource use data RCTs/RWE/observational studies/letters/case reports reporting on clinical events which may be associated with HCRU	Reviews/editorials/commentaries SLRs/(N)MAs*^a^* In vitro/animal studies/pre-clinical studies
Date limits	2003 to present*^b^*	Pre-2003
Countries	No restriction	—
Languages	English language publications	Non-English language publications

Abbreviations: CT, computed tomography; HCRU, healthcare resource use; MRI, magnetic resonance imaging; NMA, network meta-analysis; PICOS, population, intervention, comparator(s), outcomes, and study design; RCT, randomized controlled trial; RWE, real-world evidence; SLR, systematic literature review; TIO, tumor-induced osteomalacia.

^*a*^Relevant SLRs/NMAs were included at title/abstract screening stage so their bibliographic reference lists could be hand-searched for relevant studies.

^*b*^A date limit of 1993 was originally considered and applied to the search strategy; however, due to the volume of evidence identified during screening, the date limit was subsequently changed to 2003.

### Data Sources

#### Electronic databases

Searches were conducted in MEDLINE Daily, In-Process & Other Non-Indexed Citations, and Epub Ahead of Print, on March 28, 2023.

#### Hand-searching—reference lists

Hand-searching was used as a supplementary measure to ensure that relevant studies were included in the targeted literature review. Bibliographic reference lists of included studies and of relevant systematic literature reviews/meta-analyses identified during the project were screened.

### Search Strategies

The database search strings identified relevant studies (full papers or abstracts) indexed in Medline. The search included free text and Medical Subject Heading (MeSH) terms. Search terms are presented in Supplementary Table S1 of the Supplementary material (19).

### Study Selection

Titles/abstracts were screened by one reviewer against predefined selection criteria. Full-text papers were screened by one reviewer against the selection criteria to ensure the methodology and results were relevant. A record was kept of all publications excluded at this stage, along with a clear justification for their exclusion (based on the predefined eligibility criteria).

The earliest full-text manuscript reporting the primary outcome for any study was designated the primary publication. Other citations for the same study were termed “linked” citations. Linked citations that offered no unique information that were superseded by either earlier or later publications were excluded during screening. Linked citations offering unique information were included.

Results from the database searches were downloaded into Covidence® and duplicates were removed. Covidence was used to manage citation screening during the title/abstract and full-text screening phases.

### Data Extraction

Data from the relevant publications were extracted by one reviewer into standardized, piloted data extraction tables (DET) in Microsoft® Excel. The information was quality checked by a second independent reviewer. The following assumptions/interpretations were made:

- Tumor size was determined by the largest dimension recorded (cm).
- Patients with impaired physical function included those who were bedridden, those unable to walk or perform activities without assistance or walking aids, and who had experienced falls.
- Biopsy of the suspected lesion was only recorded if the biopsy was obtained to diagnose TIO. Diagnostic bone biopsy referred to any bone biopsy, except for that of a suspected lesion/tumor.
- For the purpose of this review, successful tumor resection was interpreted as biochemical cure and/or symptomatic resolution. While in clinical practice, the definition of successful surgery may more strictly comprise biochemical cure only, not all included studies explicitly reported biochemistry results post-surgery. Therefore, a broader definition was used to avoid under-reporting of the success rate. The number of resection attempts was also captured, and so was whether the final resection was successful.
- Positron emission tomography (PET) scans were categorized as “PET” or “PET/CT” when no other information was provided regarding DOTATATE or fluorodeoxyglucose (FDG) agents. Single-photon emission computed tomography (SPECT) scans included sestamibi scans (eg, technetium ^99^Tc sestamibi and ^111^indium-pentetreotide scans) and SPECT/CT scans. Dual-energy x-ray absorptiometry (DEXA) scans were assessed regarding bone densitometry.
- In cases where it was unclear whether the patient had one or multiple scans/fractures, the most conservative value was selected.

Data were collected separately for case reports (studies reporting outcomes on a patient-level basis) and publications reporting outcomes at aggregate level (case series, clinical trials, observational studies). Case series referred to papers which did not report data for individual patients, but instead only presented aggregate data from a number of case reports. Patient characteristics of all the case reports were reviewed after data extraction to check for similar ages/genders/countries, and the outcomes assessed. The case reports which were identified as duplicates of other case reports were removed. Reporting of patient-level data extracted from case reports was prioritized, with information on aggregate level studies used to supplement the findings; synthesis of aggregate level studies was not possible due to inconsistent reporting of outcomes.

### Statistical Analysis

For descriptive analyses, continuous variables were summarized as mean, median, SD, and range. Categorical variables were summarized as number of observations and percentage.

Association analyses were conducted for pairs of variables to assess predictors of healthcare resource use. The following statistical tests were conducted:

- For 2 categorial variables: Fisher's exact test
- For a continuous variable and a categorical variable: Mann-Whitney U test
- For 2 continuous variables: Spearman correlation.

To minimize bias, association analyses of attempted surgical resection, successful resection, and incomplete resection were restricted to cases in which these outcomes were explicitly reported (either in the affirmative or negative).

## Results

### Characteristics of Included Studies

**Figure 1.**
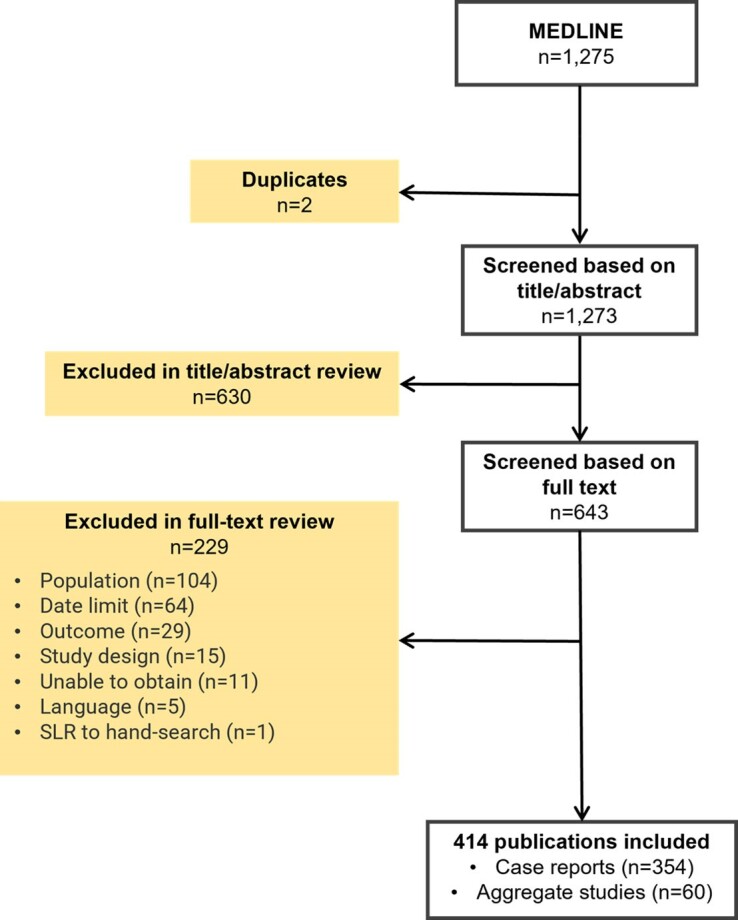
PRISMA diagram for the selection of eligible publications identified through MEDLINE.Abbreviations: PRISMA, Preferred Reporting Items for Systematic Reviews and Meta-Analyses; SLR, systematic literature review.

Figure 1 depicts the PRISMA (Preferred Reporting Items for Systematic Reviews and Meta-Analysis) diagram for the selection of eligible publications. In total, 1275 publications were identified through the electronic database searches. After the removal of 2 duplicates, 1273 publications were reviewed based on their titles and abstracts. A total of 630 publications were excluded at the title/abstract review stage, leaving 643 potentially relevant publications that were procured for full-text review. After reviewing the full-text publications, a further 229 were excluded, resulting in a total of 414 publications for final inclusion in the review. In the included publications, 44 were from 2003-2007, 84 from 2008-2012, 116 from 2013-2017, and 170 from 2018-2023. Out of the 414 included publications, 60 were aggregate studies and 354 were case reports. In 22 of the aggregate studies, case report data were identified and extracted, resulting in a total of 376 case reports for data extraction (case reports were defined as studies reporting outcomes on a patient-level basis), with a total of 621 patients. Except where otherwise indicated, the data presented relate to case report studies. A full list of included publications is presented in Supplementary Tables S2 and S3 of the Supplementary material (19). Throughout this review, the data presented are restricted to healthcare resource use explicitly reported in publications.

### Patient Characteristics

Of the 621 patients included in the case reports, the age at presentation was available for 610 patients. Mean age of patients at presentation was 46.3 years (SD 15.8) (Table 2); 34 patients (5.6%) were < 18 years old. In total, 358 patients were male (57.6%), and 263 (42.4%) patients were female. A total of 42 countries were represented, with more than half of all patients from the following countries: USA (n = 125; 20.2%), China (n = 125; 20.2%), and India (n = 108; 17.5%).

**Table 2.** Patient characteristics

Patient characteristics	
Age (years)	N = 610
Mean	46.3
Median	48
SD	15.8
Range	2-87
Age category, n (%)	N = 610
< 18 years	34 (5.6)
> 18 years	576 (94.4)
Sex, n (%)	N = 621
Female	263 (42.4)
Male	358 (57.6)
Country/Region	N = 617
USA	125 (20.3)
China	125 (20.3)
India	108 (17.5)
Europe*^a^*	93 (15.1)
Japan	72 (11.7)
Asian region*^b^*	37 (6.0)
American region (excluding USA)*^c^*	23 (3.7)
New Zealand/Australia	22 (3.6)
Middle East*^d^*	10 (1.6)
South Africa	2 (0.3)

Abbreviations: UK, United Kingdom; USA, United States of America.

^*a*^Includes: Austria, Belgium, Finland, France, Germany, Greece, Ireland, Italy, Lithuania, The Netherlands, Poland, Portugal, Slovakia, Spain, Switzerland, and the UK.

^*b*^Includes Hong Kong, Malaysia, the Philippines, Singapore, South Korea, Taiwan, Thailand, and Vietnam.

^*c*^Includes Brazil, Canada, Chile, Hawaii, and Mexico.

^*d*^Includes Iran, Israel, Jordan, Saudi Arabia, and Turkey.

### Investigations, Diagnosis, and Localization

The most commonly reported symptom at presentation was pain (n = 478; 77.0%), followed by weakness (n= 245; 39.5%), a history of fracture(s) (n = 194; 31.2%), and impaired physical function (n = 187; 30.1%) (Table 3). In patients under the age of 18 years (n = 34), lower limb deformities were reported in 10 patients (29.4%), and rickets was reported in 9 patients (26.5%).

**Table 3.** Symptoms at presentation, time to TIO diagnosis, and other attributed conditions

Description	
Symptoms at presentation, n (%)	N = 621
Pain	478 (77.0)
Weakness	245 (39.5)
History of fracture(s)	194 (31.2)
Impaired physical function	187 (30.1)
Fatigue	38 (6.1)
Height loss	28 (4.5)
Lower limb deformities	10 (1.6)
Rickets	9 (1.5)
Time from onset of symptoms to TIO diagnosis (years)	N = 422
Mean	4.6
Median	3
SD	4.7
Range	0.04-38.00
% of patients within each diagnostic interval stratum	
< 1 year	35 (8.3)
1.0-3.0 years	192 (45.5)
3.1-5 years	90 (21.3)
> 5 years	105 (24.9)
Other attributed conditions (in >1 patient), n (%)	N = 69
Osteoporosis	14 (20.3)
Ankylosing spondylitis	8 (11.6)
Arthritis/osteoarthritis	7 (10.1)
Rheumatoid arthritis	5 (7.2)
Fibromyalgia	3 (4.3)
Polymyalgia rheumatica	2 (2.9)
Tendonitis/tendon inflammation	2 (2.9)

The mean time from symptom onset to diagnosis (defined as biochemical diagnosis of TIO, and not necessarily tumor localization) was 4.6 years (n = 422; SD: 4.7) (Table 3). Mean time from symptom onset to diagnosis was highest in India (5.6 years, SD: 6.6), lowest in the Other Asia/Pacific region (including Japan, Australia, and New Zealand; 4.0 years, SD: 3.6) and similar across the American region (4.5 years, SD: 4.7), Europe/Middle East/Africa (4.5 years, SD: 4.4), and China (4.3 years, SD: 3.5).

Other conditions attributed as causes were reported for 69/621 patients (11.1%). In those patients, the most frequent conditions were osteoporosis (n = 14/69; 20.3%), ankylosing spondylitis (n = 8/69; 11.6%), and arthritis/osteoarthritis (n = 7/69; 10.1%) (Table 3). In the 34 patients under the age of 18, there were 5 (14.7%) who had other conditions attributed as a cause of their symptoms, with the following diagnoses made for 1 patient each: transient synovitis, bronchitis, growing pains/bilateral patellofemoral syndrome and bilateral pes planus, Osgood-Schlatter disease, and juvenile idiopathic arthritis. None of those diagnoses were reported in patients over the age of 18.

#### Aggregate level studies

A total of 15 aggregate level studies reported mean time from symptom onset to diagnosis (range of means, 2.4-10 years). Additionally, 2 aggregate level studies described numbers of physician visits: Hidaka et al, 2022 (20) reported the number of physicians visits before measurement of FGF23, which ranged from 0 to 8 (median: 2), and Jerkovich et al, 2021 (4) reported the number of physicians visits before diagnosis of TIO, which ranged from 2 to 15 (median: 7).

Of the 8 aggregate level studies which reported an initial diagnosis other than TIO accounting for the presenting symptomatology, 7 found that in over 80% of patients, features were initially attributed to non-TIO diagnoses (overall ranging from 11.8% to 100%). The commonly attributed conditions were aligned with those seen in the case report studies, such as osteoporosis and ankylosing spondylitis. In one of the studies, Feng et al, 2017 (21), a total of 137/144 (95.1%) patients were diagnosed with intervertebral disc herniation, spondyloarthritis (including ankylosing spondylitis), osteoporosis, femoral head necrosis, hyperparathyroidism, rheumatoid arthritis, arthritis, bone metastases, connective tissue diseases, osteoarthritis, or fibromyalgia syndrome.

#### Investigations

The following laboratory investigations were reported: serum phosphorus (n = 582, 93.7%), FGF23 (n = 398; 64.1%), alkaline phosphatase (AlkP; n = 392; 63.1%), 1,25-dihydroxyvitamin D (1,25D; n = 297, 47.8%), ratio of tubular maximum reabsorption rate of phosphate to glomerular filtration rate (TmP/GFR; n = 172; 28.0%), urine phosphate (n = 148; 23.8%), tubular reabsorption of phosphate (TRP; n = 106; 17.1%), and fractional excretion of phosphate (FeP; n = 18; 2.9%) (Table 4). In total, 313 patients (50.4%) had at least one urinary phosphate measurement. Notably, of the 223 patients without a reported FGF23 measurement, almost half (n = 106, 47.5%) were from China, accounting for 84.8% of all Chinese patients (106/125 patients).

**Table 4.** Disease investigations

Investigations	
Laboratory investigations, n (%)	N = 621
Serum phosphorus	582 (93.7)
FGF23	398 (64.1)
AlkP	392 (63.1)
1,25D	297 (47.8)
TmP/GFR	172 (28.0)
Urine phosphate	148 (23.8)
TRP	106 (17.1)
FeP	18 (2.9)
Selective venous sampling for FGF23, n (%)	61 (9.8)
Imaging procedures	N = 621
Mean number of procedures	4.1
Median number of procedures	3
SD	2.7
Range	1-17
Type of imaging procedure, n (%)	N = 621
MRI	355 (57.8)
CT	288 (46.9)
X-ray	222 (36.2)
Technetium bone scan	197 (32.1)
Octreotide scan	153 (24.6)
DEXA scan	137 (22.3)
SPECT	53 (8.6)
Ultrasound	51 (8.3)
PET scans	
DOTATATE/DOTANOC PET/CT	232 (37.8)
FDG PET/CT	135 (22.0)
PET/CT*^a^*	39 (6.4)
PET*^a^*	32 (5.2)
Biopsy, n (%)	N = 621
Biopsy of suspected lesion	122 (19.7)
Diagnostic bone biopsy	52 (8.4)

Abbreviations: 1,25D, 1,25-dihydroxyvitamin D; AlkP, alkaline phosphatase; CT, computed tomography; DEXA, dual-energy X-ray absorptiometry; FDG, fluorodeoxyglucose; FeP, fractional excretion of phosphate; FGF23, fibroblast growth factor 23; MRI, magnetic resonance imaging; PET, positron emission tomography; SPECT, single-photon emission computed tomography; TmP/GFR, ratio of tubular maximum reabsorption rate of phosphate to glomerular filtration rate; TRP, tubular reabsorption of phosphate.

^*a*^Isotope agent not reported.

The use of diagnostic bone biopsy was reported in 52 patients (8.4%), and biopsy of a suspected lesion in 122 patients (19.7%) (Table 4).

Overall, biochemical indices or biopsies were reported in 585 (95.8%) patients. For those in which these tests were not reported, the majority (21/26 patients, 80.8%) were from case report studies in China, suggesting that there is some geographical variation in the reporting of biochemical indices and biopsies, as previously noted with FGF23 measurements.

Imaging procedures were reported in 597 patients (96.1%), and the mean number of reported imaging procedures per patient was 4.1 (n = 597; SD: 2.7). The most commonly reported imaging tests were magnetic resonance imaging (MRI; n = 355; 57.8%), CT scan (n = 288; 46.9%) DOTATATE/DOTANOC PET/CT scan (n = 232; 37.8%), x-ray (n = 222; 36.2%), and bone scan (n = 197; 32.1%) (Table 4). More than one method of imaging was reported in 491 patients (79.1%). In addition to imaging, 61 patients (9.8%) had selective venous sampling for FGF23 to localize their tumor. Patients in Europe/Middle East/Africa had the highest mean number of imaging procedures (5.4, SD: 3.1), followed by those in the American region (4.9; SD: 3.0). The lowest mean number of procedures was in India (3.0, SD: 1.9), followed by China (3.2; SD: 2.0); patients in the Other Asia/Pacific region had a mean of 4.0 imaging procedures (SD: 2.3).

### Disease Characteristics

**Figure 2.**
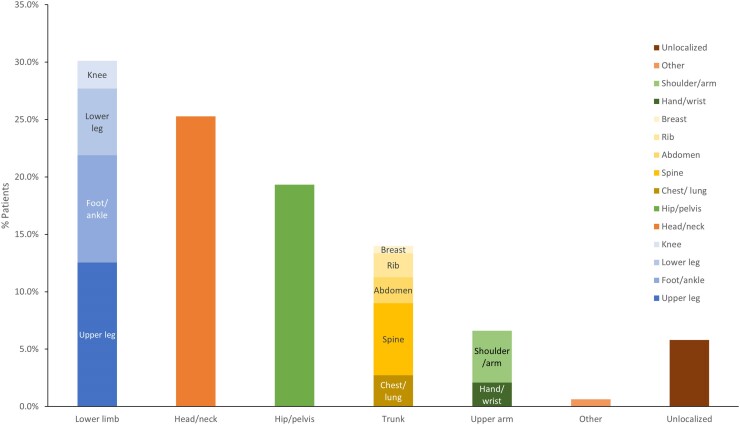
Visual representation of tumor location by anatomical region and individual site. In the case report studies, tumor locations were reported by site, which are further grouped by anatomical region here.

Tumor(s) were successfully localized in the majority of patients (n = 585; 94.2%). The most commonly reported tumor locations, by anatomical region, were the lower limb (n = 187; 30.1%), head/neck (n = 157; 25.3%), the hip/pelvis (n = 120; 19.3%), and the trunk (n = 87; 14.0%) (Table 5 and Fig. 2). Presence of a metastatic tumor was reported in 27 (4.4%) patients, of which 15 (2.4%) were metastatic at initial diagnosis. The average reported tumor size was 3.1 cm (n = 284; SD: 2.5).

**Table 5.** Disease characteristics

Tumor characteristics	
Tumor location, n (%)	N = 621
Lower limb	187 (30.1)
Upper leg/femur	78 (12.6)
Foot/ankle	58 (9.3)
Lower leg/tibia/fibula	36 (5.8)
Knee	15 (2.4)
** **Head/neck	157 (25.3)
Hip/pelvis	120 (19.3)
Trunk	87 (14.0)
Spine	39 (6.3)
Chest/lung/rib	30 (4.8)
Abdomen	14 (2.3)
Breast	4 (0.6)
** **Upper limb	41 (6.6)
Shoulder/arm	28 (4.5)
Hand/wrist	13 (2.1)
** **Other*^a^*	4 (0.64)
No location identified	36 (5.8)
Metastatic tumor	
Yes	27 (4.4)
Tumor size (cm)	N = 283
Mean	3.1
Median	2.3
SD	2.5
Range	0.5-15.5
% of patients within each tumor size stratum	
<1.5 cm	56 (19.8)
1.5-2.5 cm	101 (35.7)
2.6-4.0 cm	65 (23.0)
>4.0 cm	61 (21.5)

^*a*^Other locations included fat and bone metastases.

### Treatment

#### Tumor removal

**Figure 3.**
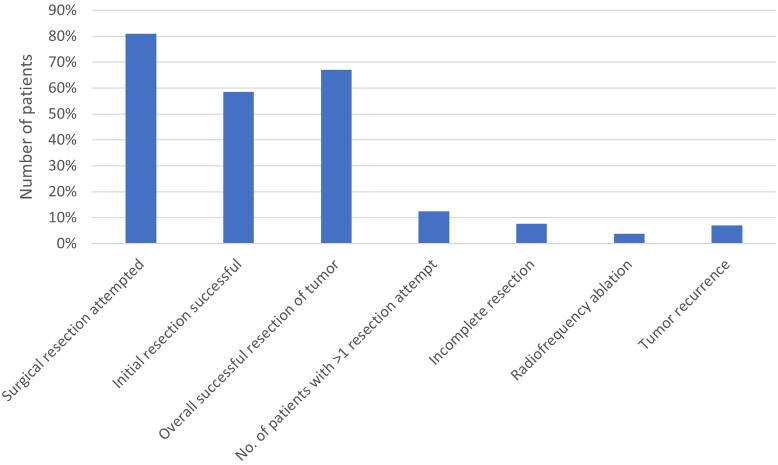
Tumor removal and recurrence. Visualization of surgical outcomes and incidence of tumor recurrence from the case report studies data.

Attempted surgical resection was reported in 503/621 patients (81.0%), and radiofrequency ablation (RFA) was reported in 24/621 patients (3.9%) (Table 6 and Fig. 3). For patients in whom resection was attempted and the number of procedures recorded, the mean reported number of resection attempts was 1.2 (n = 452; SD: 0.5); 56/452 (12.4%) patients underwent more than one surgical resection attempt. The rate of attempted surgical resection was highest in China (95.2%), followed by Europe/Middle East/Africa (88.3%). Rates of attempted resection were similar across the American region (75.7%), the Other Asia/Pacific region (75.6%) and India (73.2%).

**Table 6.** Treatment of disease

Type of treatment	
Removal of lesion	N = 621
Surgical resection attempted, n (%)	503 (81.0)
Initial resection successful	364 (58.6)
Overall successful resection of tumor, n (%)	416 (67.0)
Incomplete resection	47 (7.6)
No. of resection attempts	
n	452
Mean	1.2
Median	1
SD	0.5
Range	1-5
No. of patients with > 1 resection attempt, n (%)	56 (12.4)
Radiofrequency ablation, n (%)	24 (3.9)
Tumor recurrence, n (%)	44 (7.1)
Pharmacologic treatment	N = 621
Oral phosphate	249 (40.1)
Active vitamin D (calcitriol, etc.)	163 (26.3)
Calcium	103 (16.6)
Over-the-counter vitamin D (cholecalciferol, etc.)	90 (14.5)
Pain medication	42 (6.8)
Cinacalcet	7 (1.1)
Burosumab*^a^*	6 (1.0)
Complications associated with oral phosphate and active vitamin D*^b^*	
Hyperparathyroidism	19 (3.1)
Parathyroidectomy	7 (1.1)
Kidney stones	1 (0.2)

^*a*^Does not include clinical trial populations.

^*b*^Complications associated with, not necessarily caused by, oral phosphate and active vitamin D treatment.

Overall, successful resection (surgery resulting in biochemical cure and/or symptomatic resolution, regardless of the number of resection attempts) was reported in 416 patients (67.0%), while initial surgery was successful in 364 patients (58.6%). The rate of successful resection was highest in China (82.4%), followed by Europe/Middle East Africa (76.2%), and lowest in India (55.6%) and in the Other Asia/Pacific region (57.3%). In the American region, the rate of successful resection was 65.5%. Tumor recurrence was reported in 44 patients (7.1%); of those, 2 (4.6%) underwent radiofrequency ablation in addition to surgery. Data on duration of follow-up duration since surgery were available for 290 patients (46.7%). Mean time of follow-up after surgery was 1.5 years (SD: 2.0).

Metastatic tumors (defined as those that were metastatic upon initial diagnosis or at any time thereafter) were associated with a lower probability of attempted resection (surgical resection attempted in 80.0% of metastatic tumors and 97.6% of nonmetastatic tumors; *P* = .002). In the event of an attempted resection, metastatic tumors were associated with a higher number of resection attempts (mean of 1.8 and 1.1 attempts in metastatic and nonmetastatic tumors, respectively; *P* = .001), were less likely to be successfully resected (successful resection in 40.0% of metastatic tumors and 92.2% of nonmetastatic tumors; *P* < .001), and were associated with a higher rate of recurrence compared with nonmetastatic tumors (25.9% vs 6.2%, respectively; *P* = .002).

Resection was more likely to be attempted in patients presenting with larger tumors (mean greatest diameters were 3.2 and 1.5 cm for tumors with and without attempted resection, respectively; *P* = .009). Smaller tumors were more likely to be successfully resected (mean greatest diameter of 3.0 and 4.2 cm for successfully and unsuccessfully resected tumors, respectively; *P* = .017). Longer time to initial diagnosis (in patients who had their tumor localized) was associated with a higher number of resection attempts (*r_S_* = 0.16; *P* = .003), a lower probability of successful resection (mean time to diagnosis of 4.0 years and 6.7 years in patients with successful and unsuccessful resection, respectively; *P* = .026), and a higher probability of tumor recurrence (mean time to diagnosis of 6.1 and 4.5 years in patients with and without tumor recurrence after resection, respectively; *P* = .015). Successful resection was less likely in tumors located in the trunk (successful resection in 81.0% of tumors located in the trunk vs 91.9% of tumors in other locations; *P* = .010); resection was more likely to be attempted in cases of lower limb tumors (attempted in 100.0% of lower limb tumors vs 95.5% of tumors in other locations; *P* = .004).

#### Pharmacologic treatment

With regard to pharmacologic treatment, use of oral phosphate was reported in 249 patients (40.1%), active vitamin D/calcitriol in 163 patients (26.3%), calcium in 103 patients (16.6%), over-the-counter vitamin D/cholecalciferol in 90 patients (14.5%), pain medication (including opioids) in 42 patients (6.8%), cinacalcet in 7 patients (1.1%), and burosumab in 6 patients (1.0%) (Table 6).

In terms of complications associated with oral phosphate and active vitamin D, hyperparathyroidism was reported in 19 patients (3.1%) and was explicitly described as a possible complication of oral phosphate and active vitamin D treatment in 8/19 patients (Table 6). Of the patients with hyperparathyroidism, 7 (1.1%) underwent a parathyroidectomy. Kidney stones were reported in 1 patient (0.2%), with no reported cases of nephrocalcinosis.

Patients who did not undergo an attempted resection were more likely to receive pharmacologic treatment with oral phosphate and/or active vitamin D (43.5% of patients with attempted resection vs 81.3% of those without attempted resection; *P* = .004). Additionally, patients in whom resection was not successful were more likely to receive oral phosphate and active vitamin D (59.1% of those with unsuccessful resection vs 41.9% with successful resection; *P* = .037).

#### Aggregate levels studies

In the aggregate level study reported by Feng et al, 2017 (21), a proportion of patients were treated for other attributed conditions, such as:

- Lumbar disc herniation: physiotherapy, acupuncture, anti-inflammatory drugs, glucocorticoids, or surgery for intervertebral disc
- Spondyloarthritis: nonsteroidal anti-inflammatory drugs (NSAID), sulfasalazine, methotrexate, glucocorticoids, thalidomide, tripterygium wilfordii, etanercept, infliximab, leflunomide, physiotherapy, or acupuncture
- Osteoporosis: calcium plus vitamin D, calcitonin, or bisphosphonates

### Fractures, Surgical Procedures, and Mobility Device Use

Across the case report studies, fractures were reported in 306 patients (49.3%); specific fracture locations were reported in 214 (34.5%) patients (Table 7). For these patients, the mean number of fractures was 2.8 (SD: 2.8). The most common sites of fracture by individual site were the rib (n = 96; 44.9%), the hip (n = 84; 39.3%), and the femur (n = 64; 29.9%).

**Table 7.** Fracture characteristics

Description	n (%)
Patients with a reported fracture, N = 621*^a^*	306 (49.3)
Number of fractures, N = 214*^b^*	—
Mean	2.8
Median	2
SD	2.8
Range	1-24
Site of fracture—n (%)	
Trunk	151 (70.6)
Rib	96 (44.9)
Spine	55 (25.7)
Lower limb	128 (59.8)
Femur	64 (29.9)
Foot/ankle	36 (16.8)
Tibia/fibula	24 (11.2)
Knee	4 (1.9)
** **Hip/pelvis	121 (56.5)
Pelvis	37 (17.3)
Hip	84 (39.3)
Upper limb	35 (16.4)
Shoulder/arm	31 (14.5)
Hand/wrist	4 (1.9)
Neck/head	4 (1.9)

^*a*^Among those with fractures reported at any stage of the disease, not just in the period leading to diagnosis of TIO.

^*b*^Patients with recorded location of fracture.

In total, 71 patients were reported to have had one or more orthopedic surgical procedures. Surgical fixation of fracture was reported in 36 patients (5.8%), orthopedic spinal surgery in 6 patients (1.0%), hip arthroplasty in 23 patients (3.7%), knee arthroplasty in 3 patients (0.5%), osteotomy in 1 patient (0.2%), and other orthopedic procedures in 11 patients (1.8%).

Orthopedic surgery was more likely to be reported in patients who had a higher number of resection attempts (mean of 1.3 and 1.1 attempts in those with and without a history of orthopedic surgery, respectively; *P* = .006). Additionally, orthopedic surgery was more likely to be reported in patients with incomplete resection (reported in 25.0% and 7.7% of patients with incomplete resection, vs those with complete resection, respectively; *P* = .016).

Regarding mobility device use, use of a wheelchair or mobility scooter was reported in 60 patients (9.7%), and use of crutches, a cane, or walker was reported in 59 patients (9.5%). Twenty-seven patients (4.3%) were reported to be bedbound or having been bedbound at some point.

## Discussion

This review assessed healthcare resource use associated with TIO. A number of literature reviews have been conducted in TIO previously (22-26), but to our knowledge, this is the first focusing specifically on the resource burden.

Our review demonstrates that there is a substantial healthcare resource use associated with TIO, with particular regard to tests and procedures utilized to diagnose the condition and to localize tumors, tumor removal, treatment of fractures, and pharmacologic treatment. Additionally, the targeted literature review found that TIO is frequently associated with a substantially prolonged time to diagnosis; analysis of case reports found a mean of 4.6 years between first appearance of symptoms and correct diagnosis. Aggregate level studies reported a large number of physician visits occurring before diagnosis of TIO, indicating a considerable resource burden associated with delayed diagnosis. A relatively small proportion of patients from the case report studies were categorized as having been misdiagnosed (10.5%); however, in aggregate level studies, most reported that disease findings were initially attributed to conditions other than TIO in over 80% of patients, suggesting that misdiagnosis was underestimated in the case reports. Feng et al, 2017 (21) also reported that misdiagnosis leads to patients receiving improper treatment, potentially further increasing the resource burden. It must be noted that in instances where common conditions were diagnosed in the setting of TIO, such as osteoarthritis, it is difficult to distinguish misdiagnosis from concurrently existing conditions.

Results from association analyses demonstrated that a longer time to diagnosis was associated with poorer outcomes, including a greater number of resection attempts, a lower probability of successful resection, and a higher probability of tumor recurrence. Thus, timely diagnosis is likely to improve outcomes for patients and prevent additional healthcare resource use. Furthermore, metastatic tumors were associated with a lower probability of attempted resection than that seen with solitary lesions. In cases where resection was attempted, metastatic tumors, in comparison to solitary lesions, were associated with more resection attempts, were less likely to be successfully resected, and were more likely to recur. These findings are consistent with the nature of metastatic disease, but once again support the need for a timely diagnosis, as tumors may become metastatic over time. Unfavorable tumor resection outcomes were also associated with other forms of resource use: tumor recurrence and a greater number of resection attempts were associated with the performance of more imaging tests, while more resection attempts and unsuccessful resections were associated with greater use of pharmacologic treatment with oral phosphate and vitamin D. Additionally, more resection attempts and incomplete resection were associated with a greater likelihood of orthopedic surgery. These results support the importance of successful tumor removal to avoid the potential downstream consequences of delayed diagnosis on patient health, and the resulting additional healthcare resource use.

Association analyses also showed that resection was more likely to be attempted in larger tumors; however, smaller tumors were more likely to be successfully resected. This could be explained by the fact that larger tumors are easier to locate, whereas smaller tumors are easier to resect, once identified. Furthermore, successful resection was less likely to occur in tumors located in the trunk, and resection was more likely to be attempted in lower limb tumors, indicating poorer resection outcomes for tumors in more complicated-to-treat locations.

Outcomes of this review highlight the importance of a prompt diagnosis (likely to be facilitated by a combination of thorough biochemical evaluation and functional imaging tests, and referral to a specialist center experienced in the treatment of rare metabolic bone disease) followed by timely resection of the causative tumor, if possible. As well as reducing healthcare resource utilization associated with imaging and visits to multiple specialists while seeking diagnosis, optimization of the diagnostic and treatment pathway for TIO should result in higher cure rates, avoid repeated resection attempts, and reduce healthcare resource utilization associated with management of progressive morbidities, including treatment of fractures, orthopedic procedures, and pharmacologic treatment. An optimized treatment pathway should also reduce the loss of gainful employment and burden on caregivers, both of which impact earnings and impose a societal cost. Increasing disease awareness and educating healthcare providers on the presentation of TIO should facilitate the optimization of diagnosis and disease management.

Regarding new treatments for TIO, burosumab is approved in several countries for use in patients where the tumor cannot be localized or curatively resected. In clinical trials, burosumab treatment restored phosphate homeostasis, with improvements in skeletal health, functional mobility, and patient-reported pain, fatigue, and health-related quality of life (as assessed by the 36-item Short Form Health Survey [SF-36] v2) (3, 27). An exploratory mixed-methods analysis of some clinical trial participants treated with burosumab provided descriptions of symptomatic improvement and its clinical meaningfulness, including physical function, participation in activities of daily living, and mental well-being (28). Notably, few patients were treated with burosumab in the studies included in this review, likely due to its recent availability. As the use of burosumab in patients with TIO increases, the frequency and pattern of health care resource use among these patients may change.

Our study has several limitations. First, direct information on healthcare resource use or costs associated with TIO is sparse, likely due to the small patient population. Therefore, in many instances, resource burden must be inferred from available data (eg, fracture incidence, time from symptoms to diagnosis, number, and type of testing). Secondly, this study likely underestimates the true resource burden of TIO, as studies may not report all resource use incurred. This is supported by a previous study (18) which reported higher resource use based on medical histories of participants in a clinical trial. In particular, the current study may underestimate numbers of imaging tests and fractures: in many cases it was unclear whether the patient had one or multiple scans/fractures. In these instances, the most conservative value was selected. Additionally, the impact of repeated laboratory testing of multiple analytes over the course of diagnosis and management, as well as of medication costs over time, could not be estimated. However, this study does provide an indication of the magnitude of resource burden associated with TIO. With regard to reported clinical outcomes, the mean duration of follow-up since surgery was relatively short (1.5 years), which may have contributed to underdetection of clinical events such as tumor recurrence, of complications associated with oral phosphate and active vitamin D treatment (such as hyperparathyroidism), and underestimation of the number of orthopedic procedures. Due to variation in reporting, it was only possible to synthesize studies reporting patient-level data (without making considerable assumptions), and therefore, a substantial number of patients were excluded from the main analysis.

In addition, information on the impact of TIO on productivity is sparse. The published reports included in this analysis lacked sufficient information to draw conclusions on the impact of TIO on productivity in these patients. However, it is reasonable to expect a substantial productivity burden based on evidence in other rare diseases (29).

Publications included in the review may be subject to bias, thus limiting generalizability. For instance, case reports may be more likely to focus on noteworthy cases, and cases without confirmed tumor localization may be less likely to be published. This may explain the high localization rate in our study (94.2%), which is greater than the localization rates reported in recent cohort studies by Hoong et al, 2024 (72.0%) (30) and Pal et al, 2019 (83.3%) (31). However, there is considerable variation across publications, with other studies reporting higher localization rates of up to 100%, depending on the type of imaging test used (32, 33).

The publications included in our review were from several different countries and, therefore, data are likely to be heterogeneous due to differences across healthcare systems, access, and patient populations. Additionally, while every effort was made to check for and remove duplicates of case reports, there is no guarantee that the same patients were not included more than once in the selected publications.

Finally, although this review only included patients with a reported (rather than suspected) diagnosis of TIO, it was not possible to guarantee a definitive diagnosis in all cases. Due to a lack of standardized reporting in case studies, it was not practical to base diagnosis on outcomes of a specific combination of tests or investigations. Generally, the gold standard for diagnosis of TIO is detection of the causative tumor, confirmed by histology and/or biochemical improvement after its resection (11). To establish a reliable diagnosis, the authors recommend a combination of comprehensive biochemical evaluation (including urine/serum phosphate and creatinine, alkaline phosphatase, parathyroid hormone, 25-hydroxyvitamin D, 1,25-dihydroxyvitamin D, FGF23, and ratio of maximum rate of tubular phosphate reabsorption to glomerular filtration rate [TmP/GFR]), physical examination, assessment of family history, and functional imaging tests (including somatostatin receptor imaging), consistent with global guidance on the recognition, diagnosis and management of TIO by Jan de Beur et al, 2023 (11).

## Conclusion

TIO is associated with a substantial healthcare resource burden. As delays in diagnosis and larger tumor size are associated with poor outcomes, improvements in the diagnostic process could lead to better management of TIO, thereby benefiting patients and reducing the resource burden associated with the disease. Further research in this area would be beneficial, such as a systematic assessment of healthcare resource use in a large cohort of patients, based on medical history data or prospective follow-up. Additional research into the productivity impact of TIO in both patients and their caregivers would also be beneficial, as data on the productivity burden of TIO are limited.

## Data Availability

Some or all datasets generated during and/or analyzed during the current study are not publicly available but are available from the corresponding author on reasonable request.

## Abbreviations

CT: computed tomography
FGF23: fibroblast growth factor 23
MRI: magnetic resonance imaging
PET: positron emission tomography
SPECT: single-photon emission computed tomography
TIO: tumor-induced osteomalacia
